# Looking for Calcium Phosphate Composite Suitable to Study Osteoclast Endocytosis: Preliminary Observations

**Published:** 2016-05-16

**Authors:** V Nicolin, G Baldini, D De Iaco, R Bortul, G Turco, SL Nori

**Affiliations:** 1 Clinical Department of Medical, Surgical and Health Science, University of Trieste, Strada di Fiume 447, 34149 Trieste; 2 Department of Medicine – University of Salerno, Via S Allende, Baronissi, Salerno

**Keywords:** biomaterial, calcium phosphate, type I collagen, monocytes, macrophages, osteoclasts, adhesion, phagocytosis, bone matrix resorption

## Abstract

One of the issues regarding in vitro study of bone resorption is the synthesis of a bone-like biomaterial forming a thin layer onto either glass or plastic. The synthesis of a bone-like material suitable for in vitro studies can be valuable both to investigate osteoclast differentiation, that in vivo proceeds within the local microenvironment of bone and to understand how its presence triggers activation of macrophages present in situ when bone is damaged (a scenario that can occur for example in case of bone fracture). Despite the intensive studies committed to recreate synthetic bone analogues, the most used substrates for in vitro studies on bone resorption are slices of bone or dentine. Therefore morphological investigations (i.e. fluorescence analysis and phase contrast) are strongly compromised due to the thickness of the bone analogue.

In the present study, with the aim to guarantee a versatile (and easy to be made) substrate, that could be suitable to study cell adhesion and morphology by epifluorescence, phase contrast and TEM, we developed a biomaterial containing a calcium phosphate salt and type I collagen. This material (made specifically for in vitro studies) forms a very thin layer that allowed to merge the morphological information derived from phase-contrast and epifluorescence observation, making possible the observation of the interface between cell and matrix.

Moreover the electron microscopy evaluation of the endocytosis performed on cell differentiated could be more suitable because sample does not need the process of demineralization.

## I. INTRODUCTION

Bone tissue is a highly dynamic structure that is renewed through a remodelling process involving osteoblasts and osteoclasts. [1,2] Due to pathological conditions such as trauma or tumours, bone defects appear, and autologous bone graft is the gold standard for bridging these gaps. [3] However, in addition to drawbacks such as potential infection and pain at the donor site, [4,5] the availability of autologous bone is limited. This prompted the development of synthetic materials as an alternative. Nanotechnology provides new materials in the nanometer range with many potential applications in research and medicine. Due to their unique size-dependent properties, nanomaterials, such as nanoparticles, offer the possibility to develop both new diagnostic and therapeutic tools. One desirable characteristic of these materials in bone is their ability to be remodelled, i.e. that osteoclasts resorb the material and it is subsequently replaced by newly formed bone through osteoblastic activity. Bone slices, as well as dentine or ivory slices, have been extensively used as substrates to study both osteoclast actin-rich adhesions and matrix resorption. [6,7] Several studies performed in the last decades [8,9,10,11] pointed on the complexity to create biologically inspired synthesis of bone-like composites. It was previously assessed by an in vivo study [12] that nanophase HA coatings trigger a better osteointegration compared to microcrystalline HA coatings; Motskin et al. [13] have demonstrated that hydroxyapatite nanoparticles are efficiently internalized from the extracellular environment by human macrophages whereas microparticles are not. Taking together these results, our aim is to find a new composite that could be useful study the bone turnover processes on a mineral layer, reducing, at the same time, the impact of thickness of bone slices usually used.

## II. METHODOLOGY

### Composites preparation (CaP and CaP/type I collagen)

#### A. CaP preparation

Aqueous solutions of 0.1 M calcium chloride dehydrate (CaCl_2_. · 2H_2_O) and 0.06 M dibasic sodium monohydrogen phosphate (Na_2_HPO_4_ · 2H_2_O) (both from Sigma Aldrich, Saint Louis, MO, USA) were prepared freshly using Millipore water and sterilized by filtration, in agreement with a previous report. [11] The biomaterial (CaP) was obtained by adding one part of 0.1 M CaCl_2_. · 2H_2_O to one part of 0.06 M (Na_2_HPO_4_ · 2H_2_O), pH 9.3 at RT.

#### B. CaP/type I collagen preparation

One part of 5.3 mg/ml type I collagen in 0.02 M acetic acid, pH 3.67, purchased from Millipore (Temecula, CA, USA) obtained from rat tails (negative for the presence of mycoplasma, bacteria and fungi), was added to two parts of the CaP solution, prepared as above at RT to make CaP/type I collagen. Composite (CaP/type I collagen) drops are seeded and dried both on the electron microscopy grids and on the coverslips immediately after mixing.

### Cell cultures

The murine monocytic-macrophagic cell line RAW 264.7 CRL-2278 (American Type Culture Collection, Rockville, MD) was grown in RPMI-1640 supplemented with 10% foetal bovine serum in an atmosphere with 5% CO_2_, at 37°C. Subcultures were prepared by scraping.

CRL-12557, a murine osteoblast-like cell line, was cultured in alpha MEM (the alpha modification of MEM with non essential amino acids, sodium pyruvate, and additional vitamins as reported in Sigma M8042 product information) supplemented with 10% foetal bovine serum, 0.2g/l L-glutamine and 1% penicillin/streptomycin.

### Coculture

RAW 264.7 type CRL-2278 murine monocyte-macrophage cell line co-cultured with murine osteoblast type CRL-12557 has been previously used as a model of osteoclastogenesis. [14]

Cells were cultured at a density of 10^5^ cells/ml for each cell line in RPMI-1640 medium with 0.3 g/l L-glutamine and modified to a final concentration of 4.5 g/l glucose, 1.5 g/l sodium bicarbonate, 10 mM Hepes, 1 mM sodium piruvate, and 10% foetal bovine serum.

After 72 h in the dual cell culture, several (about 20%) binucleate or multinucleate cells could be observed.

### Nuclear and F-actin fluorescent staining

One drop of freshly made CaP/type I collagen was added to round glass coverslips and left to air dry under the biological safety cabinet. Sterile coverslips were placed in a 12-well plate, washed twice for 10 min with sterile PBS, then the complete culture medium previously described was added. RAW 264.7 cells and CRL-12257 osteoblasts plated together for 72 h were lifted by trypsin treatment, scraped and seeded overnight on coverslips with the biomaterial CaP/type I collagen. After 24 h, seeding coverslips were quickly washed with PBS at RT and fixed for 20 min at RT with 4% paraformaldehyde in PBS. Cells were then permeabilized with 0.3% Triton X-100 in PBS for 10 min at RT and washed three times with PBS. After washing, cells were incubated for 5 min with DAPI (Sigma) and then with 1:50 FITC-phalloidin (Molecular Probes, Eugene, OR, USA) in PBS for 30 min. Coverslips were observed with a Zeiss Axiophot fluorescence microscope (Carl Zeiss, Germany), and micrographs were collected with a CoolSnap video-camera (Roper Scientific, Inc. Tucson, AZ, USA).

### Transmission (TEM) and scanning (SEM) electron microscopy of the CaP/type I collagen composite

#### A. TEM analysis of substrate

A parlodion carbon coated grid (SPI Supplies, West Chester, PA, USA) was dipped into a drop of either CaP or CaP/type I collagen and air dried, then washed in PBS for 5 min and finally rinsed in water. The specimen (without any stain) was examined with a Jeol 100S TEM microscope (Jeol Ltd, Tokyo, Japan) at 80kV.

#### B. SEM Analysis

Coverslips with CaP/type I collagen were mounted on aluminium stubs covered with two sides conductive carbon adhesive tape. Subsequently, the samples were gold sputtered (Sputter Coater K550X, Emitech, Quorum Technologies Ltd, UK) and immediately observed by means of a SEM Quanta250 (FEI, OR, USA) operating in secondary electron detection mode. The working distance was adjusted in order to obtain the suitable magnification with an accelerating voltage of 30kV.

### TEM of cultured cells

Both RAW 264.7 monocyte cells grown as a single clone and cocultures previously described were grown for 72 h in a 10-cm Petri dish, then 600 μl of CaP/type I collagen was added.

After 18 h, cells in presence of the attached CaP/type I collagen complex were harvested and prepared for the Electron Microscopy analysis. Cells were pelleted and fixed with 2% glutaraldehyde (Electron Microscopy Sciences, Hatfield, PA, USA) in 0.1 M phosphate buffer, pH 7.3, for 1 h at RT. Post fixation was performed with 0.3 mg/ml ruthenium red in phosphate buffer for 1 h at 4°C and then with 1% OsO_4_ in 0.1 M phosphate buffer for 1 h at 4°C, dehydrated through an ascending alcohol series, treated with propylene oxide at RT and embedded in Durcupam A/M epoxy resin.

Ultrathin sections were cut using a Top Ultra 170 Reichert OM (Wien, Austria) ultramicrotome. Then counterstained with uranyl acetate and lead citrate, and finally observed using a JEOL 100S (Jeol Ltd, Tokyo, Japan) electron microscope.

## III. RESULTS

### CaP and CaP/type I collagen composite

**Fig. 1.**
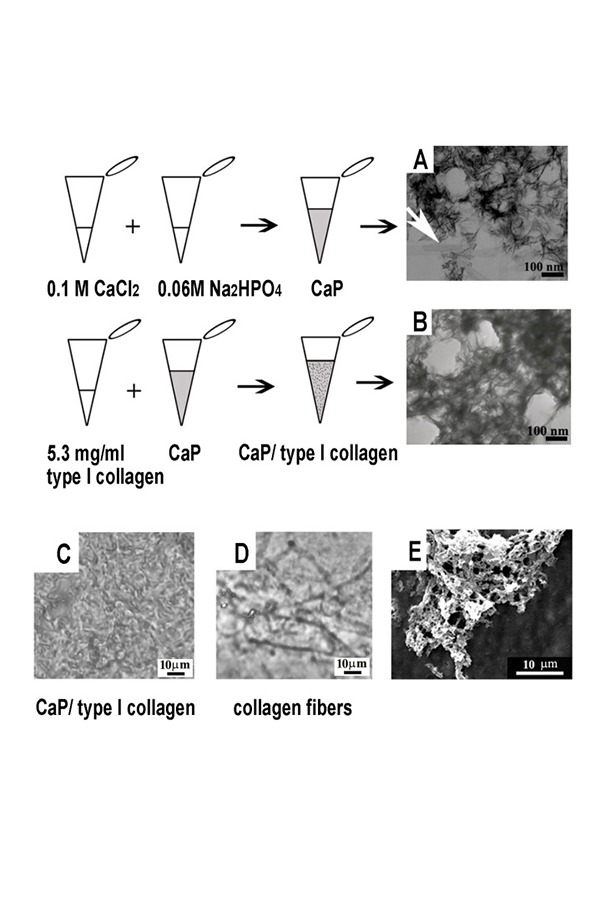
**A:** CaP is constituted by nano scaled (40–90nm), needle-like particles with the long axis oriented randomly and embedded in a scaffold of amorphous material. **B:** CaP/type I collagen has a similar texture but presents a less electrondense appearance. **C:** light microscopy, phase contrast image of CaP/type I collagen seeded on a glass slide. **D:** phase contrast image of collagen at the same concentration used in CaP/type I collagen but without calcium chloride dehydrate, dried onto a glass slide when a neutral pH is restored. Collagen forms fibers. **E:** SEM image of CaP/type I collagen.

We have mixed one part of 0.1 M CaCl_2_ with one part of 0.06 M of Na_2_HPO_4_. Calcium cations (Ca^2+^) and phosphate anions (HPO^3−^) precipitated quite immediately to form the composite CaP. The CaP substrate observed by TEM presented a structure of needle-like electrondense particles (length between 40 and 90 nm) with the long axis oriented randomly (Figure 1A). The white arrow points out the presence of some plate-like thin mineral particles of greater dimensions (length 200 nm, width 30 nm) that could be ascribed to the growth in size of some crystals. One part of type I collagen (Figure 1B) was added to the mixture named CaP (constituted by one part of 0.1 M CaCl_2_ with one part of 0.06 M of Na_2_HPO_4_). The TEM micrograph of the composite, named CaP/type I collagen, exhibited a similar nanostructure, but less electrondense.

Light microscopy phase contrast analysis revealed that CaP/type I collagen composite presented an amorphous morphology in which no collagen fibers could be recognizable (Figure 1C). When collagen, were added to one part of water mixed with one part of 0.06 M of Na_2_HPO_4_ (instead of the mixture constituted by one part of 0.1 M CaCl_2_ plus one part of 0.06 M of Na_2_HPO_4_), formed fibers as shown in Figure 1D.

### Cell adhesion and polarization of cell grown on CaP/type I collagen composite

**Fig. 2.**
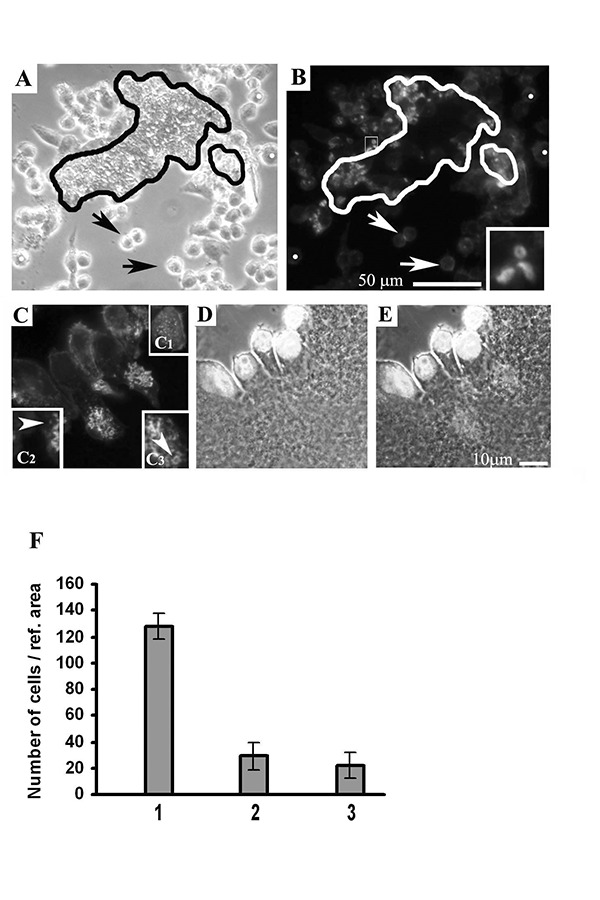
**A,B:** phase-contrast of fluorescence micrograph of the same field to compare actin fluorescence of cells near to the edge of CaP/type I collagen versus cells seeded on glass: the physical contact with the two materials elicits a different actin organization. Arrows: cells on glass, white dots: reference points on same cells. In the right corner of B a fourfold enlargement of an area is shown. **C,D:** two micrographs of the same field to describe the spatial position of actin structures, nucleus and CaP/type I collagen. The first using filters for FITC (green fluorescence), the second is taken with both transmitted phase contrast and epifluorescence lights on and DAPI (blu) fluorescence filters inserted. **E:** the two images C, D were overlapped with Adobe Photoshop 7.0 program. C1: the insert shows an undifferentiated RAW 264.7 cell cultured on glass with individual podosomes scattered in the ventral zone of the cell body. C2 and C3: two enlargements of C showing that the size of the formed actin patches are bigger than the punctate, single podosome fluorescence.

We investigated cell adhesion to the CaP/type I collagen composite adhesion of RAW 264.7 monocytes cultured in the presence of CRL-12557 osteoblasts on the CaP/type I collagen substrate occurred very rapidly, since within 1 hour monocytes adhered to the substrate, as observed by phase contrast on living cells. In Figure 2A and 2B the perimeter of the CaP/type I collagen covered area is outlined in black (Figure 2A) and in white (Figure 2B). Cell grown on the CaP/type I collagen composite revealed an early podosome organization marked by FITC/phalloidin (green) fluorescence in comparison to cells grown on glass (Figure 2B, arrows).

Even if cells presented only one nucleus (Figure 2C, D and E) acquired a polarized shape with the nucleus located on the opposite side of the contact cell-CaP/type I collagen zone. On the same side, some scattered individual podosomes were present (arrow), but the most part of polymerized actin formed patches, open lines or round shaped, adhesion structures in comparison with the dot-like individual podosome fluorescence detected on monocyte seeded on glass (Figure 2C_1_).

### Internalization process of CaP/type I collagen composite

**Fig. 3.**
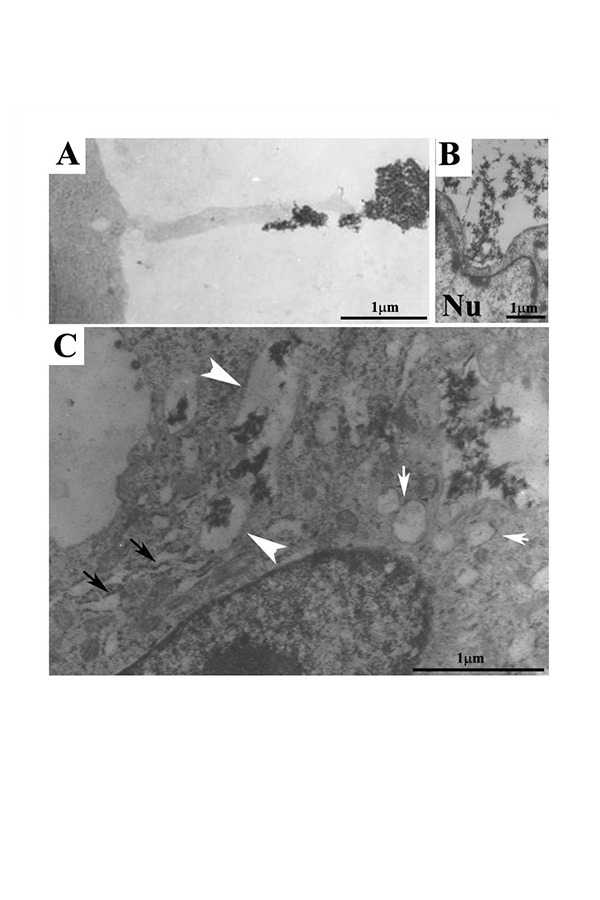
**A:** a contact exerted by a podosome of RAW 264.7 type CRL murine monocyte-macrophage with the mineralized matrix. **B:** CaP/type I collagen internalized by plasma membrane invagination by a cell presenting nuclear deformation. **C:** CaP/type I collagen is easily detected in the cytoplasm (white arrows) and is contained in large vesicles of irregular shape enveloped by a single membrane. In close spatial relation to phagosomes there is an abundant dilated rough endoplasmic reticulum (C, black arrows) and clear vesicles.

TEM observation of a macrophage that incorporates bone matrix by long cytoplasmic processes was previously reported. [15] Figure 3A shown a cytoplasm extroflection (contact cytoplasmatic extroflection) of a monocyte-macrophage cell on the CaP/type I collagen surface. Contact zone shown a finger like structure with a diameter of 0.3 μm. At the same time Figure 3B and 3C, shown CaP/type I collagen composite internalized by large plasmalemmal invaginations that could be considered an early phase of phagosome formation (as shown in Figure 3B). The presence of CaP/type I collagen complex inside the cytoplasm is observed in Figure 3C as mineral particles contained in large vesicles surrounded by a single membrane (arrowheads). An abundant dilated rough endoplasmic reticulum (Figure 3C, black arrows) is found in close spatial relation to phagosomes (Figure 3C, white arrows). Clear vesicles, with some of them fusing together, (white arrows) are also found near the phagosomes.

### CaP/type I collagen composite and cell differentiation

Cells grown on the composite presented specialized plasma membrane characteristics such as presence of the ruffled border, the typical plasma membrane area committed to degrade and resorb bone.

**Fig. 4.**
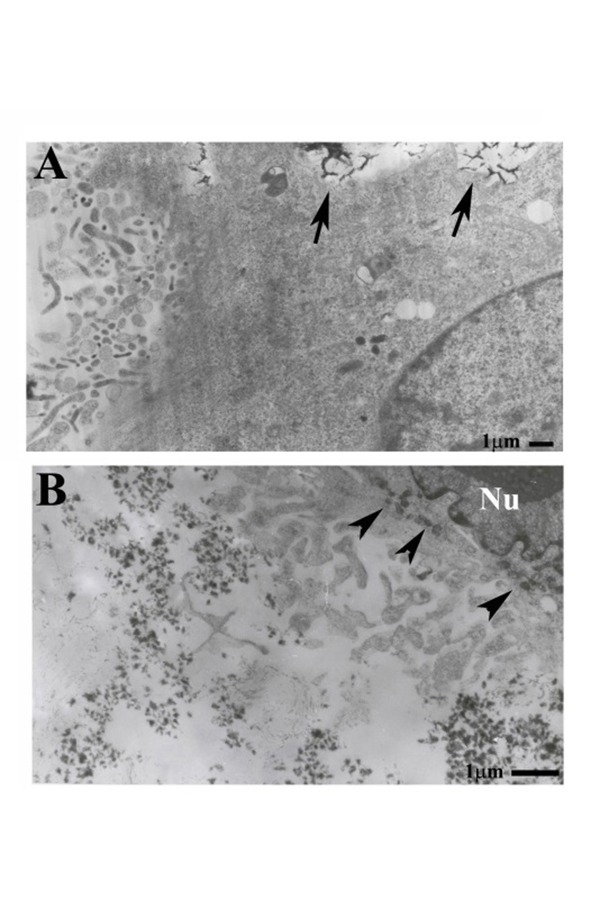
**A:** the cell in the micrograph presents both a ruffled border at the left and large vesicles of undegraded CaP/type I collagen (black arrows) which we have found also in cells that has no ruffled border and therefore are at a lower level of differentiation. **B:** CaP/type I collagen facing the ruffled border is more fragmented and numerous fine collagen nanofibrils are present (black arrows).

The cell in Figure 4A presents both a ruffled border (at the left side of the micrograph) and large phagosomes (arrows) contained undegraded CaP/type I. This is consistent with previous findings that in vitro osteoclasts induced degradation is performed by simultaneous resorption and phagocytosis mechanism. [16]

CaP/type I collagen facing the ruffled border is more fragmented and numerous nanofilaments could be detected (Figure 4B). Several electrondense bodies are observed (Figure 4B, arrowheads) inside the cell.

## IV. DISCUSSION

The mineralized tissues, *i.e.* bone and dentin, contain hydroxyapatite (HA) crystals with an irregular shape inside collagen fibrils that function as a mold to the crystal growth. [17,18] Therefore, the best technique to form in vitro a structure resembling the bone matrix is to grow HA crystals inside a layer of collagen fibrils so that the crystal dimensions, orientation and density are identical to those found in actual bone. Intuitively the most reliable method to obtain such as substrate would be a bioinspired mineralization of collagen.

[19] Olszta et al. have proposed a model of in vivo mineralization where a liquid amorphous phase precursor of the HA crystals infiltrates the collagen fibrils.

The liquid phase mineral precursor, which is able to reach the nanoscopic gaps inside collagen fibrils through capillary action, is believed to transform itself into oriented crystals of apatite after settling into the interior of the collagen fibrils. [19] This hypothesis is supported by Tampere *et al*. [8] actually in vitro mineralization with reconstituted HA crystals that come into contact with type I collagen leads to the deposit of HA crystals on fibers, but HA does not penetrate within the fibrils and there is no real interaction of HA with collagen fibers. But the *in vitro* synthesis of a stable amorphous mineral liquid phase necessary as precursor to nucleate and growth crystals inside collagen fibrils is still under study; Desponded *et al*. [20] have obtained mineralized fibrils with the presence of poly L-aspartic acid (used as a model polyelectrolyte) that inhibits mineralization in a concentration dependent manner obtaining a bio inspired synthesis of mineralized fibrils. On the other hand some interesting methods to mineralize collagen fibrils without the aid of polyamine polymers were performed: Tampere *et al*. [8] (method 2) use a phosphoric acid solution containing collagen dropped in a calcium hydroxide aqueous solution or Maas *et al*. [11] pump the acidic solution of calcium and collagen through a nanoporous membrane to a receiver solution containing phosphate anions. As recently reported [21] collagen fibrils with the ordered periodic 67nm cross-striated structure provide a template that induces oriented apatite nucleation. Despite these efforts, the constitution of bone-like mineralized fibrils is far from following an easy protocol and even if intrafibrillar mineralization is achieved it seems very difficult to obtain the high mineral amount attained biologically by intrafibrillar mineralization. Our aim was to create a mineralized surface that could be used to study the bone turnover and endocytosis processes inside the cells, avoiding problems caused by thickness of surfaces usually used.

The composite that we have proposed, presented many differences between other materials previously used such as collagen fibrils are not present and mineral particles are embedded in tropocollagen matrix. Although the Ca/P ratio used is within the range used by Maas *et al*. [11], the CaP/type I collagen ratio is one thousand times greater.

As assessed by electron microscopy, CaP is constituted by needle-like particles randomly oriented that strongly resemble hydroxyapatite crystals, and the addition of collagen does not alter the morphology of the CaP structure which remains the same except for diminished electron density.

The highly ordered mineralization at the nanoscale that is responsable for the biomechanical properties of bone mineralized collagen [22] is not present in CaP/type I collagen, however, the nanoscale dimension of mineral particles are similar to those present in bone. In fact the length of the nanoparticles in CaP is almost identical to the length of the needle-like crystals described by others [17] in the turkey tendon collagen fibers; in the CaP/type I collagen the presence of tropocollagen confers to the matrix a composition similar to bone. The orientation of the needle-like particles in our CaP/type I collagen is random; possibly because the collagen present in our composite does not form fibrils and therefore there is not a template for oriented crystallization.

Osteoclasts, grown on our composite, develop different adhesion structures according to their substratum [23,24] underlines the importance of a substrate that mimics bone material and therefore can be used for in vitro studies of cells that interact with bone. Moreover the major problem regarding the study of bone resorption, at an ultrastructural level, is the need to demineralised the bone in order to cut the ultrathin sections for electron microscopy. Unfortunately demineralization leads to a decrease of the minerals electron density in the extracellular space and possibly inside the cells, making the mineralized elements less recognizable (maintaining a high electron density of the mineral particles is of great importance for TEM analysis of bone dissolution by resorptive cells). The electron microscopy reveals the presence of CaP/collagen inside the cells, confirming that the composite is internalized.

The Tem analysis clearly shown the relationship between the endoplasmic reticulum, phagosomes and clear vesicles, suggesting that phagosomes originated by internalization of CaP/type I collagen. Some authors have investigated the degradation of calcium phosphate ceramics exerted by monocytes suggesting that monocytes could internalize the biomaterial by phagocytosis and subsequently the material undergoes on dissolution inside the cytoplasm. [24]

Our results confirm their findings concerning the process of mineral incorporation by intracytoplasmatic phagocytosis. Moreover it was assessed that macrophage internalization of mineral particles leads the production of many mediators that could stimulate the activity of osteoclast cells. [25] In more differentiated cells we have shown that the presence of CaP/type I collagen induces, like bone surfaces, the formation of ruffled borders, the complex system of plasma membrane invaginations facing the acidic compartment where resorption takes place, [26] which is a characteristic of active, resorbing osteoclasts. Previous observations, confirmad by our ultrastructural observations, shown that osteoclasts exerted some phagocytosis processes during degradation of calcium phosphate ceramic. In conclusion, in this study we have proposed a mineral easy made surface that could be suitable for optical and electron microscopy.

Even if the presence of CaP/type I collagen creates less difficulties in ultrathin sectioning in comparison with bone because the mineral particles of the composite are not densely packaged as the mineral particles in bone.

The CaP/type I collagen composite showed good potential for clinical applications as it was shown biocompatible. These basic properties show good perspectives for other studies focused on its mechanical properties and osteoinduction, These future prospectives could be useful to develop new scaffold for tissue engineering.

## Abbreviations

TEM: transmission electron microscopy
SEM: scanning electron microscopy
ER: endoplasmic reticulum
RT: room temperature
HA: hydroxyapatite
